# B cell receptor repertoire analysis from autopsy samples of COVID-19 patients

**DOI:** 10.3389/fimmu.2023.1034978

**Published:** 2023-02-23

**Authors:** Sadahiro Iwabuchi, Tomohide Tsukahara, Toshitugu Okayama, Masahiro Kitabatake, Hideki Motobayashi, Shigeyuki Shichino, Tadashi Imafuku, Kenzaburo Yamaji, Kyohei Miyamoto, Shinobu Tamura, Satoshi Ueha, Toshihiro Ito, Shin-ichi Murata, Toshikazu Kondo, Kazuho Ikeo, Yutaka Suzuki, Kouji Matsushima, Michinori Kohara, Toshihiko Torigoe, Hiroki Yamaue, Shinichi Hashimoto

**Affiliations:** ^1^ Department of Molecular Pathophysiology, Institute of Advanced Medicine, Wakayama Medical University, Wakayama, Japan; ^2^ Department of Pathology, Sapporo Medical University School of Medicine, Hokkaido, Japan; ^3^ Laboratory of DNA Data Analysis, National Institute of Genetics, Shizuoka, Japan; ^4^ Department of Immunology, Nara Medical University, Nara, Japan; ^5^ Second Department of Surgery, Wakayama Medical University, Wakayama, Japan; ^6^ Division of Molecular Regulation of Inflammatory and Immune Disease, Research Institute for Biomedical Sciences, Tokyo University of Science, Chiba, Japan; ^7^ Department of Diseases and Infection, Tokyo Metropolitan Institute of Medical Science, Tokyo, Japan; ^8^ Department of Emergency and Critical Care Medicine, Wakayama Medical University, Wakayama, Japan; ^9^ Departments of Human Pathology, Wakayama Medical University, Wakayama, Japan; ^10^ Department of Forensic Medicine, Wakayama Medical University, Wakayama, Japan; ^11^ Department of Computational Biology, Graduate School of Frontier Sciences, The University of Tokyo, Chiba, Japan; ^12^ Departments of Cancer Immunology, Wakayama Medical University, Wakayama, Japan

**Keywords:** COVID-19, B cell receptors, lung, single cell, serum IgGs, vaccine intake

## Abstract

Neutralizing antibodies against the severe acute respiratory syndrome coronavirus 2 (SARS-CoV-2) are being developed world over. We investigated the possibility of producing artificial antibodies from the formalin fixation and paraffin-embedding (FFPE) lung lobes of a patient who died by coronavirus disease 2019 (COVID-19). The B-cell receptors repertoire in the lung tissue where SARS-CoV-2 was detected were considered to have highly sensitive virus-neutralizing activity, and artificial antibodies were produced by combining the most frequently detected heavy and light chains. Some neutralizing effects against the SARS-CoV-2 were observed, and mixing two different artificial antibodies had a higher tendency to suppress the virus. The neutralizing effects were similar to the immunoglobulin G obtained from healthy donors who had received a COVID-19 mRNA vaccine. Therefore, the use of FFPE lung tissue, which preserves the condition of direct virus sensitization, to generate artificial antibodies may be useful against future unknown infectious diseases.

## 1 Introduction

Although the coronavirus disease (COVID-19) pandemic caused by severe acute respiratory syndrome coronavirus 2 (SARS-CoV-2) has subsided, the pandemic is still raging in some countries owing to insufficient vaccine supply and the emergence of mutant SARS-CoV-2 strains. Randomized clinical trials of mRNA-based vaccines have reported up to 95% efficacies in the prevention of COVID-19 (1). Recombinant monoclonal antibodies generated by the B cell receptor (BCR) repertoire of patients have been useful for treating the respiratory syncytial virus infection (2). Rearrangement of the BCR encoding genes induces recombination of variable (V), diversity (D), and joining (J) segments of the third complementarity determining region (CDR3), leading to considerable diversification. Understanding the diversity of BCRs, their response to SARS-CoV-2 infection, and detection of specific BCRs may help in the development of therapeutic antibodies for patients with COVID-19. The life cycle of SARS-CoV-2 has been revealed (3), and multiple studies have investigated the development of neutralizing monoclonal antibodies targeting the SARS-CoV-2 spike (S) protein (4–12). Candidate antibodies are usually obtained by analyzing BCRs in the peripheral blood mononuclear cells (PBMCs) obtained from patients with COVID-19 compared with those from healthy donors (HDs). Although the number of candidate antibodies against SARS-CoV-2 reported from multiple institutions is constantly being updated, the virus is constantly mutating; therefore, a combination of two or three neutralizing antibodies that can target different “weak” spots on the virus is more effective than a single neutralizing antibody. Casirivimab and Imdevimab (REGEN-COV) are the cocktail of two neutralizing antibodies that targets the receptor-binding domain (RBD) of the S protein to prevent binding to the S antigen, ACE2 (13, 14). These antibodies have been rationally designed to bind distinct and non-overlapping regions of the RBD, resulting in simultaneous blockage (13).

We had provided an overview of RNA expression, immune cell populations, cytokine expression, and histopathological characteristics of the formalin fixation and paraffin-embedding (FFPE) lung lobes of a patient with COVID-19 (15, 16), and the comprehensive analysis indicates the distribution of SARS-CoV-2 and the cellular and molecular differences among mild-to-severely inflamed microenvironment in different lung lobes. We further found that the severely inflamed lung lobes highly express aquaporin-3 (AQP3)-positive basal-like cells and alveolar type II cells, which proliferate abnormally to fill the alveolar space and stromal tissue that collapses upon SARA-CoV-2 infection (15). The B-cells and plasma cells existed in the inflamed lung lobes; therefore, we hypothesized the presence of antibodies with neutralizing activity against SARS-CoV-2 more in the lung lobes.

Here, BCR repertoire analysis was performed using a unique method than the conventional approach by analyzing of BCRs in FFPE lung lobes from the same patient and we developed several artificial antibodies using pairs of IgG heavy and light chains, which were frequently detected in the inflamed lung lobe. To realize the significance of the detected BCR repertoires, single-cell BCR (scBCR) repertoires obtained from the PBMCs of HDs who had recovered from COVID-19 or had received the mRNA vaccine or had not received the vaccine were also analyzed. Moreover, we evaluated SARS-CoV-2 neutralizing activity along with the artificial antibodies, and it was increased by mixing several artificial antibodies than each of them alone. The results of this study shed light on the development of vaccines and neutralizing antibodies using FFPE samples against future unknown infectious disease.

## 2 Methods

### 2.1 Samples and findings

Detailed information about the patient has been described previously (16). A 79-year-old man was admitted to our intensive care unit with respiratory failure due to COVID-19. After a 16-day course of invasive mechanical ventilation, the patient died of multiple organ failure. Fixed lung lobes, retrieved from this patient, were embedded in paraffin, to form formalin-fixed paraffin-embedded (FFPE) tissue, according to standard methods. This study was approved by the Research Ethics Committee of Wakayama Medical University (approval no.: 2882), and verbal consent was obtained from relatives to use FFPE lung tissue for research.

### 2.2 Bulk BCR sequencing and analyzing

Five tissue sections (10 μm thick) from the FFPE block in the left upper lobe (LUL) or right lower lobe (RLL) were cut, and RNA was obtained using NucleoSpin total RNA FFPE kit (Marcherey-Nagel GmbH & Co. KG, Düren, Germany) following the manufacturer’s instructions. BCR repertoire analysis was performed using the Archer Immunoverse-HS BCR IGJ/K/L kit (Invitrogen, Carlsbad, CO, U.S.A.) following the manufacturer’s protocol. In brief, RNA was transferred to BCR-specific reverse transcriptome priming tubes and incubated for 5 min at 65°C. After the first and second cDNA synthesis, dual-index sequence adaptors were attached to both ends of the strands, and sequencing libraries were obtained following amplifications. The final fragment size of the library was 203–322 bp. Sequencing was performed for samples with 10% PhiX control (Illumina, CA, U.S.A) using a NextSeq 500/550 Mid Output Kit v2.5 (Illumina; 300 cycles, 150/150 cycles, paired-end). The total sequence reads in the LUL and RLL were 88,897,047 and 45,823,220 reads, respectively. The sequencing data file was analyzed using the Archer Analysis software 6.2. All datasets were deposited under DDBJ DRA; accession number DRA013492 and BioProject accession number PRJDB13054.

### 2.3 Immunohistochemistry

Tissue sections (4 µm thick) were cut, dewaxed, and rehydrated using xylene and graded alcohol. The sections were then inactivated by treatment with an antigen activator (pH 9.0) for 20 min at 95°C, and probed with anti-CD19 (Leica Biosystems, Wetzlar, Germany, NCL-L-CD19-163, 1:100) overnight at 4°C. Thereafter, the sections were treated with mouse anti-IgG antibodies for 30 min at 20°C and visualized following treatment with 3,3-diaminobenzidine for 10 min at 20°C. Subsequently, the sections were counterstained with hematoxylin. All the stained sections were examined under a fluorescence microscope (BZ-X710; KEYENCE, Osaka, Japan).

### 2.4 Single-cell BCR repertoire sequencing and analysis

High-throughput scBCR repertoire analysis was performed using BD Rhapsody TCR/BCR profiling assays (Beckton, Dickinson and Company [BD], NJ, U.S.A.) with modifications (17). This study was approved by the Research Ethics Committee of Wakayama Medical University (approval no.: 2961). Written informed consent for participation was obtained in accordance with the national legislation and the institutional requirements. Peripheral blood (8−10 mL) in BD Vacutainer CPT tubes (BD) was centrifuged at 1,500×g for 15 min to obtain PBMCs. After hemolysis with ammonium chloride, the B cells were negatively selected using a Miltenyi magnetic-activated cell sorting bead isolation kit (Miltenyi Biotec Inc., Bergisch Gladbach, Germany) (18). A total of 350,000 cells were loaded into Nx1-seq as previously described (19), and mRNA-captured barcoded beads were used to synthesize cDNA. To modify the VDJ library amplification protocol, cDNA was amplified using human B cell PCR primers. The average library size ranged from 795 bp to 1,220 bp. High-throughput sequencing was performed on samples with a 20% PhiX control using a NovaSeq 6000 S4 Reagent Kit v1.5 (Illumina; 300 cycles, 75/225 cycles; paired-end). The analysis pipelines were modified based on SevenBridges, which is an analysis pipeline for BD Rhapsody assays (17). The fastq data were validated by the identification methods in which the error correction was equivalent to the SevenBridges, and the average of the filtered data was 87.3%. The data were subjected to a BLASTN (version 2.9.0+) search against the IMGT database (https://www.imgt.org/) using the R2 sequence corresponding to each barcode in the R1 sequence as the query. The top five results per query (=1 read) were tabulated along with the barcode of the R1 sequence. The data that exceeded a certain threshold were omitted, i.e., if the number of queries exceeded the number of cells loaded into the Nx1-seq. For each of the heavy (V-D-J-C) and light (V-J-C) chain patterns, the data were aggregated as single or a pair in a frequency distribution. The percent ratio of total BCR immunoglobulin heavy (IgH) or light (IgK) chains was calculated; for example, a total of 23,565 BCR IgH chains were detected and the top 100 frequently detected BCR IgH chains were 7,516 in HD no. 1 who had recovered from COVID-19. All the sequenced BCR IgHs (20,565) included only one count that was detected, and it did not seem to be an important IgH. Therefore, we set the threshold above the highly detected top 100 BCR IgH chains. In this case, the most frequently detected IgH was 818 counts, and the 100^th^ was 25 counts. *IGHV3-23-IGHD1-IGHJ4-IGHG2*, *IGHV3-23-IGHD2-IGHJ4-IGHG2*, and *IGHV3-23-IGHD1-IGHJ5-IGHG2* were detected in 818, 598, and 90 of total 7,516 IgH chains, respectively, indicating that the total ratio (%) of selected *IGHV3-23* for the top 100 IgH chains was 20.0%. Computations were partially performed on the NIG supercomputer at RIOS National Institute of Genetics. All datasets were deposited under DDBJ DRA accession number DRA013491 and BioProject accession number PRJDB13013.

### 2.5 Plasmid construction and IgG production

The pFUSE-Fc plasmid (400 mg) was supplied by the Department of Pathology, Sapporo Medical University School of Medicine. The retroviral reprogramming plasmids pFUSE-hIgG1-Fc2 (InvivoGen, CA, U.S.A.) have been previously described (21). pFUSE-Fc2 (IL2ss) plasmids facilitate the secretion of Fc-Fusion proteins from pFUSE-Fc-transfected cells. To obtain single-chain Fc fragments of IgH and IgK, we followed the principle of PCR assembly (22), and the information of the inserted synthesized representative cDNA and the amplified primers are shown in **Supplementary Table 1**. Briefly, the plasmid was digested with EcoRV and NcoI (Takara Bio, Shiga, Japan), and the targeted IgH and IgK domains were obtained using a two-step overlapping PCR. The PCR product was subcloned into the digested plasmid using In-Fusion HD Cloning kits (Takara Bio); the In-Fusion reaction mixture was transformed into competent cells, and individual isolated colonies were picked from the culture plate. Plasmid DNA was isolated using a Plasmid DNA purification kit (Macherey-Nagel GmbH& Co.), and 2.5 μg of the DNA transfected into 293 T cells using Lipofectamine 3000 (Thermo Fisher Scientific, MS, U.S.A.). Subsequently, the 293 T cells were grown in Dulbecco’s Modified Eagle Medium containing 10% fetal bovine serum and 1% penicillin-streptomycin solution with zeocin (300 μg/mL, InvivoGen), and the supernatant was collected from the culture well on day 2 and days 7–10. IgGs from the supernatant were collected from 3–5 culture dishes and purified using a spin column-based antibody purification kit (Protein G; Cosmo Bio, Tokyo, Japan). The density of IgG was measured using the NanoDrop One (Thermo Fisher Scientific).

### 2.6 Screening scFv specifically reacting with human SARS-CoV-2 spike protein using the scFv phage display library derived from naïve donors

Isolation of single-chain Fv fragments (scFv) clones specifically reacting with the human SARS-CoV-2 spike protein was performed according to our previous report with some modifications (23). Biotinylated human SARS-CoV-2 spike protein (#HAK-SPD _BIO-1, Hakarel Co., Ltd., Ibaraki, Japan) and biotinylated human ACE2 protein (#AC2-H82E6, AcroBiosystems, Inc., DE, U.S.A.) were used as antigens. First, the biotinylated ACE2 protein was mixed with the scFv phage display library constructed from naïve donors to remove scFv reacting with the ACE2 protein non-specifically (negative panning). Subsequently, the resultant library was mixed with SARS-CoV-2 protein to enrich for specific scFv (positive panning). After two-rounds of negative and positive panning, soluble scFv expression in *Escherichia coli* infected with the phage was induced. The resulting supernatant was immediately used for enzyme-linked immunosorbent assay (ELISA) screening. One scFv clone that reacted with SARS-CoV-2 but not with ACE2 was isolated. Sequences containing the IgH region, peptide linker, and IgK region are shown in **Supplementary Data Sheet 2**.

### 2.7 Neutralization assay for SARS-CoV-2

To evaluate the neutralization effect of SARS-CoV-2, we utilized the SARS-CoV-2 Neutralization Antibody Detection Kit (MBL, Tokyo, Japan) and SARS-CoV-2 Neutralization Ab ELISA Kit (Invitrogen) following the manufacturer’s protocol. Briefly, we extracted and purified IgG (100 μg/mL) from each sample and applied 10 μg to the RBD of an ACE2-coated reaction plate. The plate was then incubated for 2 h at 24°C. The IgG in the plasma specimens from seven HDs was purified using a spin column-based antibody purification kit (Protein G; Cosmo Bio), and the density of IgG was measured using a NanoDrop One (Thermo Fisher Scientific). A positive control (10 μg) was added to each kit. After washing the ACE2 reaction solution (His-tagged human ACE2 protein), 100 μL of horseradish peroxidase-conjugated anti-His-tag monoclonal antibody was added and incubated for 30 min at 24°C. The absorbance of each sample at 450 nm was measured using a microplate reader. A 600 nm wavelength filter was used as a reference. The measured values in the blank, positive control, and each sample were often indistinguishable, which could be due to the long-term storage of the kits, albeit at the fridge temperature in lightproof containers, or the use of kits with different lot numbers. Therefore, we duplicated the measurements of each artificial antibody and IgG with a positive or negative control for each experiment. To eliminate any experimental errors due to differences in the measurement conditions or product lots, we measured each sample at least three times. To verify the performance of the SARS-CoV-2 neutralization activity kits, we measured the activity using two different kits and calculated the mean ± standard error (S.E) in each group. The inhibition rate (%) was calculated using the following equation [1].


[1] $$\text{Inhibition rate }\left(\%\right)=\frac{\left(\text{O}.\text{D}.\text{ value of Blank}\right)-\left(\text{O}.\text{D}.\text{ value of Sample}\right)}{\left(\text{O}.\text{D}.\text{ value of Blank}\right)-\left(\text{O}.\text{D}.\text{ value of Positive}\right)}\times 100$$

The IgGs obtained from 3–5 different culture conditions were measured at least three times using the neutralization kit (MBL) twice to confirm the variations in data due to differences in the product lot. The neutralization assay was repeated using a different kit (Invitrogen). A total of 8–16 samples were measured for each condition, and the average inhibition rate was calculated. In some cases, the optical density (O.D.) value of the sample was lower than that of the positive control.

A second assay was performed to evaluate the neutralizing antibody using the plaque reduction neutralizing test (PRNT) with live viruses (20, 24). The SARS-CoV-2 S protein (319–541 aa) monoclonal antibody for neutralization (0.1–100 μg; Catalog #67758-1, Proteintech, IL, U.S.A.) was incubated with equal volumes of 100 plaque-forming units of SARS-CoV-2 (nCoV-19/JPN/TY/WK521/2020, National Institute of Infectious Disease, Japan) at 37°C for 1 h. Half of the virus-serum mixture was then infected with VeroE6/TMPRSS2 cells (JCRB1819, JCRB Cell Bank, Japan) at 37°C for 1 h and covered with agarose overlay. After 48 h of incubation, the cells were fixed with 10% formalin and stained with crystal violet. Virus-infected cells are lysed and form crystal violet-negative holes (plaques); hence, the effect of neutralizing activity was calculated by counting the number of plaques. The plaque count in the negative control groups was 49.5–65.0, and the 50% reduction of the plaque number (number of plaques< 25–32.5) in the positive control (SARS-CoV-2 S protein) was more than 10 μg. Therefore, we applied the same amount (10 μg) of purified IgG from each sample to the assay and calculated the neutralization activity (%). SARS-CoV-2 infection experiments were conducted twice in at least 2–3 culture dishes to ensure reproducibility.

### 2.8 Statistical information

**Figure 1:**
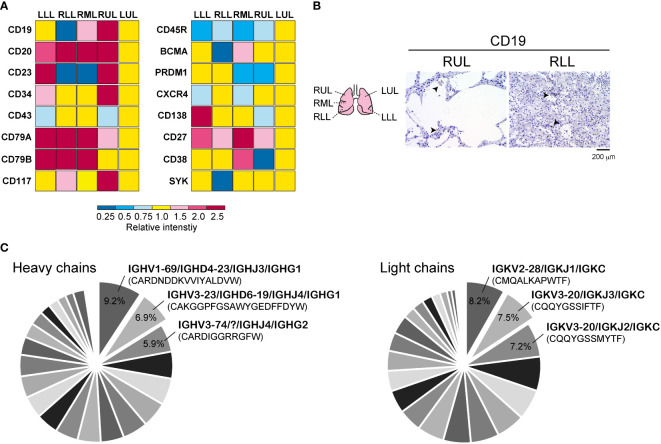
Detection of B cell-related genes in autopsied lung tissue. **(A)** Representative mRNA distributions of the B cells in each lung lobe by analyzing bulk RNA-seq data. **(B)** Immunohistochemistry was performed by using CD19 antibody in right upper lung lobe (RUL) and right lower lung lobe (RLL). Schematic diagram of each lung tissue site (left) is shown. Scale bar indicates 200 μm. **(C)** The pie chart of B Cell receptor (BCR) heavy and light chains obtained from bulk BCR repertoire analysis in upper lung lobe. Representative IgH and IgK chains with corresponding CDR3 sequences are presented. The percent ratio for all detected BCR repertoires is shown. All raw data are presented in **Supplemental data 1**.

We conducted a within-subjects analysis of variance (ANOVA) on the conditions, following Welch’s t-test, and *p*< 0.05, which showed a significant difference between the conditions in **Figure 1**.

## 3 Results

### 3.1 Identified specific B cell repertoire in lower lung lobes

The representative mRNA for B cells in the bulk RNA-seq data from each site of the lung lobe are shown in **Figure 1A**. The expression of the biomarker for normal and neoplastic B cells, *CD19* mRNA (25), varied among each site of the lung lobe. *CD20* mRNA, which is expressed on the surface of normal and malignant B cells (26), was increased in the lower lung lobes. In addition, the expression levels of the memory B cell marker, *CD27* mRNA, and the positive regulators of BCR signaling, *CD79a* and *CD79b* mRNA (27), were relatively higher in the lower lung lobes compared with left upper lobe (LUL). Immunostaining for CD19 showed that B cells remained relatively intact in the upper lobes where the alveoli were preserved, but were present as singles or in clusters with diverse shapes in the highly inflamed lower lobes (**Figure 1B**). A comprehensive bulk-based analysis of a diverse repertoire of BCRs from the LUL tissues was performed to identify the specific IgH and IgK genes expressed in response to SARS-CoV-2 infection (**Figure 1C**). The detection sensitivity of the BCR repertoire in FFPE samples was considerably low, and the total counts of IgH or IgK per 4,306,115 sequencing reads in the LUL specimens were recorded. Subsequently, 36 IgH genes and 28 IgK genes were identified in the LUL tissues (**Supplementary Data Sheet 2**). *IGHV1-69/IGHD4-23/IGHJ3/IGHG1* and *IGHV3-23/IGHD6-19/IGHJ4/IGHG1* accounted for 9.2% and 6.9% of the total detected IgH chain genes in LUL, respectively, whereas *IGKV2-28/IGKJ1/IGKC* and *IGKV3-20/IGKJ3/IGKC* accounted for 8.2% and 7.5% of the total detected IgK chain genes, respectively (**Figure 1C**). Although the types of (D) and (J) of CDR3 were different, the heavy chains, *IGHV1-69* and *IGHV3-23*, were detected in 9.2% and 12.5% of the total, respectively, whereas the light chains, *IGKV2-28* and *IGKV3-20*, accounted for 31.3% and 23.7% of the total, respectively (**Supplementary Data Sheet 2**). In 25%–30% of the LUL regions, we observed mildly thickened alveolar walls and hyaline membrane formation with mild inflammation and diffused alveolar damage, whereas the lower lung lobes showed fibrous and thickened alveolar walls with severe inflammation (15). Therefore, it was difficult to identify multiple IgH genes from the RLL samples, as the sequencing library was excessively fragmented (**Supplementary Figure 1**); therefore, only two IgH genes and 18 IgK chain genes were identified in the RLL tissues (**Supplementary Data Sheet 2**). However, 40% of the total detected IgK chain genes in RLL were *IGKV2-28/IGKJ1/IGKC*, and its expression levels were the highest in LUL.

### 3.2 Single-cell B cell receptor repertoire in PBMC from three categories of HDs

**Figure 2:**
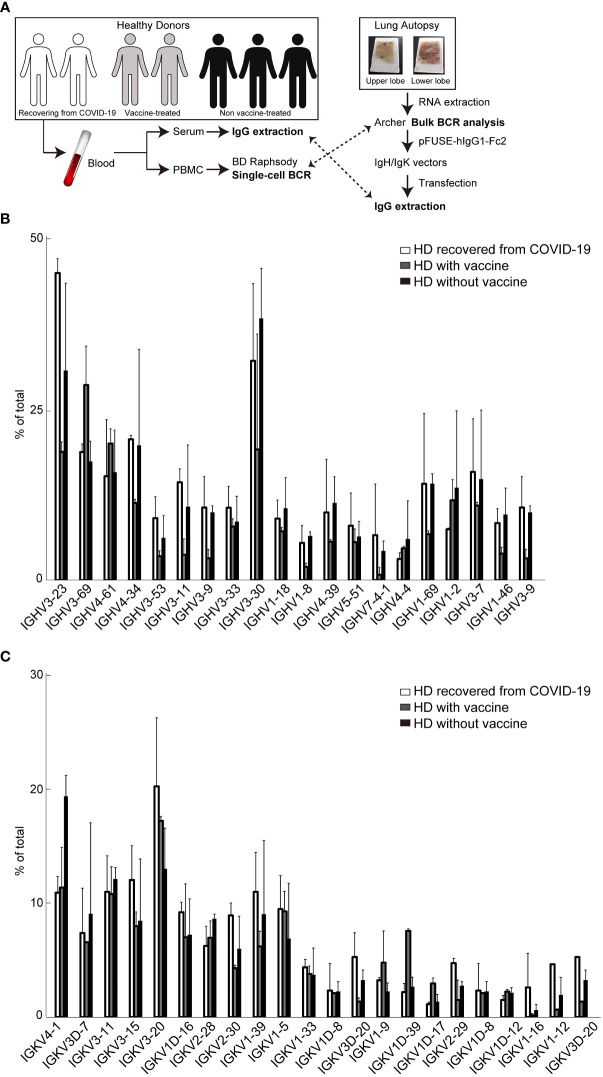
Single-cell B cell receptor (scBCR) repertoire analysis in peripheral blood mononuclear cells (PBMC) -derived B-cells purified from whole blood of each donor. **(A)** Schematic of the experiment. The distribution of representative BCR IgH **(B)** or IgK **(C)** chains detected from seven healthy donors (HDs) is shown. These graphs indicate the average ratio (%) of total BCRs (top 30,000) in each group; two HDs recovered from COVID-19 (white bar), two HDs with vaccine treatment (grey), and three HDs without vaccine treatment (black). The bars indicate mean ± standard deviation (S.D.).

**Figure 3:**
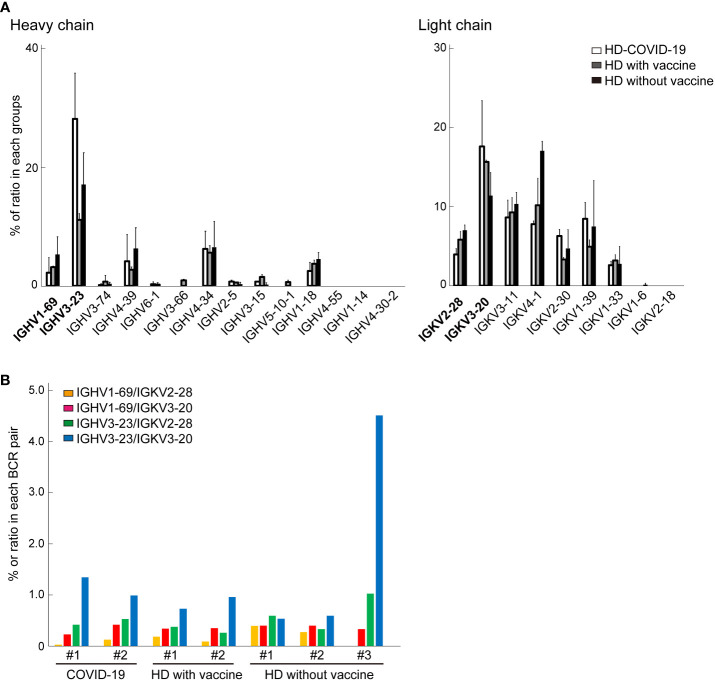
The comparison of B cell receptors (BCRs) from bulk BCR analysis in Formalin-fixed paraffin embedded (FFPE) lung tissue and single cell BCR (scBCR) analysis. **(A)** The distribution of representative BCR IgH or IgK chains detected from lung tissue in healthy donors (HDs) is shown. These graphs indicate the ratio (%) of total BCRs in each group; HDs recovered from COVID-19 (white bar), HDs with vaccine (grey), and HDs without vaccine treatment (black) groups. The bars indicate mean ± standard deviation (S.D.). **(B)** The graph shows the % of the top two most detected pairs of BCR IgH and IgK chains from lung tissue for scBCR repertories in each group.

Next, we performed scBCR sequencing (scBCR-seq) of two HDs who recovered from COVID-19 (HD-COVID-19) at least six months prior, two HDs who had been treated with an mRNA vaccine (mRNA-1273 or BNT162b2) twice at least one-two months prior (26 or 57 days after 2^nd^ vaccine treatment; HDs with vaccine) and three HDs without vaccine (HDs without vaccine) (**Figure 2A**). The trial was being conducted in the early 2020 and the first dose of the vaccine was still awaiting its turn in Japan. To determine SARS-CoV-2 infection status, serum samples from the seven HDs were measured using SARS-CoV-2 IgG antibody test kit and an iFlash 3000 chemiluminescence immunoassay analyzer (28–30). The detection values (AU/mL) of N and S proteins for SARS-CoV-2 in the HD-COVID-19 groups were above the threshold (5 AU/mL), and those of S1 protein in the HDs with vaccine group were high, indicating that the serum utilized in this experiment showed the state of recovery after natural infection with SARS-CoV-2 and after injection of the mRNA vaccine incorporating S protein (**Supplementary Table 2**). Although there was no significant difference in the expression levels of BCR IgH and IgK chains in each group, the expression of *IGHV3-23* in the HD-COVID-19 group and *IGHV3-69* or *IGKV1D-39* in HDs with vaccine group tended to be slightly higher (**Figures 2B, C**). The results of BCR pairs in each group are shown in **Supplementary Figure 2**, and paired identification analysis of BCR IgH and IgK chains showed that diversity existed among the donors. To investigate how many of the BCRs obtained from FFPE lung lobes were expressed in the PBMCs of HDs, we calculated the percentage ratio for each detected chain in each group (**Figure 3A**). The expression of *IGHV1-69*, which was the most highly expressed gene in FFPE samples, was detected at low levels in BCRs from PBMC. As for light chains, *IGKV4-1* tended to be slightly higher in the HDs without vaccine group; however, its expression was similar in all the groups. The sequencing results obtained by screening the scFv phage display library specifically reacting with the human SARS-CoV-2 spike protein indicated that *IGHV3-23* and *IGKV2D-28* were the candidates (**Supplemental data 1**). The pair of highly expressed IgH and IgK chains in FFPE samples (*IGHV1-69*/*IGKV2-28*, *IGHV1-69*/*IGKV3-20*, *IGHV3-23*/*IGKV2-28*, and *IGHV3-23*/*IGKV3-20*) were not specifically expressed in the PBMCs of HDs (**Figure 3B**). These results suggest that the four BCR pairs, especially *IGHV1-69* chosen from FFPE lung lobes, may be novel antibody candidates.

### 3.3 Neutralizing activity test against SARS-CoV-2

**Figure 4:**
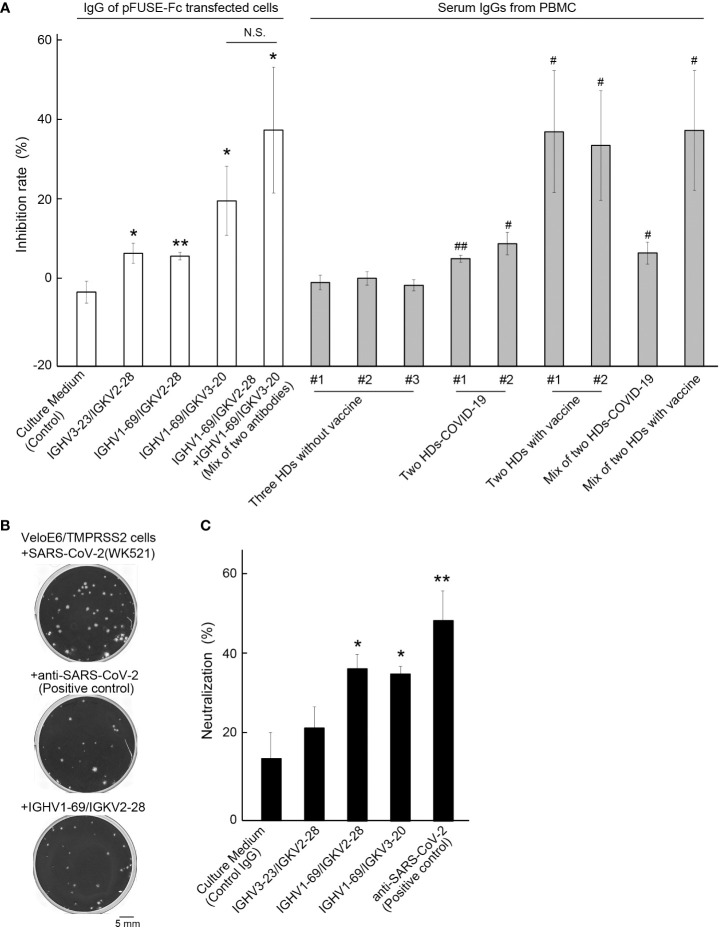
Neutralizing activity test against SARS-CoV-2. **(A)** The inhibition rates of each artificial antibody and serum-derived IgG from seven healthy donors (HDs) are shown. Control: IgG from culture media transfected with an empty vector. The numbers (#1, #2, or #3) indicate the IgG in each HD, and #1+#2 in HD-COVID-19 or #1+#2 in HD with vaccine groups represent a mixture of IgGs from two different HDs. Bars indicate the mean ± standard error (S.E.). * or **, *p*< 0.05 or 0.01 vs Control group, ^#^ or ^##^, *p*< 0.05 or 0.01 vs #1 of HDs without vaccine group. **(B)** Representative images of plaques (crystal violet-negative holes) in the negative control (upper), positive control (middle), and IGHV1-69/IGKV2-28 treatment groups (lower). Scale bar = 5 mm. **(C)** Neutralization activity of live SARS-CoV-2 in each sample is shown. The effect of neutralizing activity was calculated by counting the number of plaques, and the average % of neutralization for monoclonal anti-SARS-CoV-2 antibody (positive control) was 44.28 ± 6.28% (mean ± S.E.). The bars indicate the mean ± S.E. * or **, *p*< 0.05 or 0.01 vs Control. Control: IgG from culture media transfected with an empty vector. The number of samples in the control, *IGHV3-23/IGKV2-28*, *IGHV1-69/IGKV2-28*, *IGHV1-69/IGKV3-20*, and positive control groups were 3, 5, 4, 5, and 3, respectively. N.S., Not Significant.

We performed a SARS-CoV-2 neutralization assay for three artificial antibodies and IgG samples obtained from the plasma specimen of HDs. The same IgG concentration 10 μg of each sample was applied to two different the SARS-CoV-2 Neutralization kits. Compared to the control IgG, which was purified from the culture medium in empty plasmid-transfected HEK293 cells, the inhibitory effect of the three artificial antibodies was significantly enhanced (*p* = 0.023, 0.0026, and 0.045) (**Figure 4A**). The highest average inhibition rate was 20.18 ± 8.35% (mean ± S.E.) for *IGHV1-69/IGKV3-20*, with a maximum inhibition ratio of 102.77%. The lowest inhibition rate was 5.26% for the same artificial antibody, and the results varied even when the same concentration of IgG was collected from different cultures. Co-treatment with two different artificial antibodies enhanced the neutralization activity (37.35 ± 15.22%, mean ± S.E., *p* = 0.028). As for the IgG derived from the peripheral blood of each donor, SARS-CoV-2 suppression was significantly higher for IgG derived from HD-COVID-19 and HDs with vaccine groups. The average inhibition rates in the three HDs without vaccination (no.1; #1, no.2; #2, and no.3; #3), two HDs-COVID-19, and two HDs with vaccine groups were 0.37 ± 0.88, 7.81 ± 1.32, and 35.16 ± 0.88%, respectively (mean ± S.E.). The maximum inhibition ratios for HD-COVID-19 and HDs in the vaccine groups were 24.18% and 96.90%, respectively. However, the inhibitory effect was not enhanced by the addition of IgG from various serum sources, possibly because the amount of IgG against RBD in the neutralizing activity kit was not saturated, and the specific antibodies in each serum IgG were diluted or offset. A separate neutralization assay using live SARS-CoV-2 indicated that the neutralization ratios for control (empty plasmid), *IGHV3-23/IGKV2-28*, *IGHV1-69*/*IGKV2-28*, *IGKV1-69*/*IGKV3-20*, and monoclonal SARS-CoV-2 neutralization antibody (positive control) were 16.00 ± 4.49, 20.75 ± 6.93, 34.91 ± 5.44, 32.05 ± 2.58, and 44.28 ± 6.28% (mean ± S.E.), respectively (**Figures 4B, C**). The maximum neutralizing ratio of *IGHV1-69*/*IGHV2-28* against live SARS-CoV-2 was 55.5%, similar to that of the positive control. Three of the two artificial antibodies had the potential to block SARS-CoV-2 significantly (*p* = 0.028, 0.023). The dose-dependent neutralizing activity of the positive control and IGHV1-69/IGHV2-28 antibody is shown in **Supplementary Figure 3**.

## 4 Discussion

Recombinant monoclonal antibodies generated from the BCR repertoire of patients have been useful for treating respiratory syncytial viral infections. Understanding the diversity of BCRs, their response to SARS-CoV-2 infection, and detection of specific BCRs may aid in the development of therapeutic antibodies for patients with COVID-19. Researchers from various disciplines have identified specific BCRs related to COVID-19 (4–8, 11, 12, 27). The antibody-mediated defense against viruses has been defined in the serum, with IgG being the main immunoglobulin isotype. However, peripheral blood may not be the adequate site where antibodies directly prevent SARS-CoV-2 infection. Instead, the antibodies cooperate with antiviral substances released by the respiratory epithelium in the respiratory mucosa to capture viruses (31). A multiparametric immunomorphological analysis in the lung tissue of patients with COVID-19 revealed a strong infiltration of B cells and plasma cells without T cell infiltration, suggesting good local production of antibodies (32). In this study, artificial antibodies against IgH and IgK chains of BCR, identified in FFPE lung tissues, were developed. We hypothesized that BCRs in the lung tissue of patients who died of pneumonia caused by COVID-19 would have more direct neutralizing activity against SARS-CoV-2 than antibodies in the serum.

Several BCR IgH and IgK chains have been detected in the upper lung lobe, following signs of mild inflammation and SARS-CoV-2 detection (15). *IGHV1-69*, *IGHV3-23*, *IGKV2-28*, and *IGKV3-20*, which were the most frequently detected IgH chains (**Figure 1C**), were compared with the BCR repertoires associated with COVID-19 reported in other studies. From a list of 294 SARS-CoV-2 RBD-targeting antibodies, *IGHV3-53* is the most frequently detected antibody in samples obtained from patients with COVID-19 (9, 12). *IGHV1-69* and *IGHV3-23* were among the top 7 and 10 antibodies detected, respectively, and some of the BCR heavy chains detected in the lung lobe were also frequently detected (9, 12). Bulk BCR heavy chain analysis in the PBMCs from 19 patients showed that *IGHV4* families expression levels were higher compared with those from HDs, whereas *IGHV1-69* and *IGHV3-23* were expressed at higher levels in the HD groups (8). However, it was indicated that *IGHV1-69* was highly reactive against RBD- and *IGHV3-23* against N-terminal domain (NTD)-sorted mAbs for SARS-CoV-2. A previous study comparing PBMCs from 12 COVID-19 recovery patients and six HDs indicated that *IGHV3-23* was significantly increased in the COVID-19 group, and *IGHV1-69* was expressed at similar levels between the groups (6). Similar results have been reported using single-cell RNA-Seq of PBMCs, which showed that *IGHV3* families are expressed at high levels in patients with COVID-19 (5). In contrast, Zhou et al. indicates that *IGHV1-69* is the most frequently used IGHV gene of 54 monoclonal neutralizing antibodies established in BCRs of PBMC from three COVID-19 donors (33). In addition, BCR repertoire analysis by single-cell RNA-seq of blood samples revealed that *IGHV3-23* is highly represented both in patients and HDs (11), indicating that the response to SARS-CoV-2 might be commonly elicited after vaccination. In this study, paired PBMC-derived BCR repertoires of five convalescent COVID-19 HDs showed that the expression of *IGHV1-69*/*IGKV2-28* and *IGHV1-69*/*IGKV3-20* was weak in both patients with COVID-19 and HDs. In contrast, *IGHV3-23*/*IGKV2-28* and *IGHV3-23*/*IGKV3-20* levels were higher in both the groups. Thus, *IGHV3-23* was equally expressed in the B cells derived from the peripheral blood of patients with COVID-19 and HDs. It is unclear to what extent *IGHV1-69*, which was prominently expressed in the B cells of the lung lobe, was also expressed in the B cells in peripheral blood from the same patient since we did not perform BCR repertoire analysis in PBMC. Although the reports of BCR repertoires related to SARS-CoV-2 vary by the degree of symptoms, duration of disease onset, and race, data have been accumulated. Two representative IgH chains *IGHV1-69* and *IGHV3-23* from our BCR repertoires analysis in FFPE tissue conducted in early 2020 suggest the possibility of simulating subsequent large-scale reliable studies.

Similar to other reports, we also examined BCR repertoires in the peripheral blood of two HDs who had been infected with COVID-19 and had recovered at least six months prior (HD-COVID-19), two HDs who had been treated with an mRNA vaccine (mRNA-1273 or BNT162b2) at least two months prior (HDs with vaccine), and three HDs who had not received the vaccine (HDs without vaccine). There are five classes of immunoglobulins: IgA, IgD, IgE, IgG, and IgM, and the expression of IGHG1 and IGHG2, the subtype of IgG, tended to be higher in the HD-COVID-19 group, whereas IGHM was higher in the HDs with vaccine group (**Supplementary Figure 4**). The HD-COVID-19 group tended to express IgL subtype IGLC2, whereas the HDs with vaccine group tended to express IGLC3, and no markedly increased immunoglobulins were found in the HDs without vaccine group. Our results differed from the immunoglobulin subtype analysis of the BCR repertoire of PBMC in 16 patients with COVID-19 and eight HDs previously reported and were more similar to the expression distribution in the HDs (7). However, IGHG1 tended to be highly expressed in the patients with COVID-19. In our analysis, *IGHV3-23*, *IGKV3D-20*, and *IGKV2-29* expression levels were higher in the BCR of PBMC from the HD-COVID-19 group, while *IGHV3-69* and *IGKV1D-39* were detected in HDs with vaccine, and *IGKV4-1* in HDs without vaccine (**Figures 2B, C**). Our findings of a higher expression of *IGHV3-23* in PBMC from patients with COVID-19 are consistent with a previous report (6); however, conflicting findings have been reported elsewhere (4, 11). Wang et al. systematically performed BCR analysis in PBMCs from HDs with no history of SARS-CoV-2 infection 2 months after the third dose of mRNA-1273 or BNT162b2 mRNA vaccines (10). The IgG heavy chain, *IGHV3-53* and *IGHV3-30*, and IgG light chain, *IGKV1-39* and *IGKV1-33*, were expressed at higher levels in HDs with vaccine groups. Here, *IGHV3-53*, *IGHV3-30*, *IGKV1-39*, and *IGKV1-33* expression levels in the HDs with vaccine group were similar to others. The discrepancy may be due to the different vaccines utilized and the possibility of Japanese-specific BCR repertories. To the best of our knowledge, there have been no reports of paired PBMC-derived BCR repertoires in HDs with SARS-CoV-2 mRNA vaccination. The major BCR pairs in each sample are presented in **Supplementary Figure 2** and only one HD-COVID-19 and two HDs with vaccine tended to have a higher percentage of specific BCR pairs. The exact same BCR pairs in each group was not detected, making the interpretation difficult. The increase in BCR pairs with *IGHV3-69-1* due to vaccine treatment may be meaningful, but further studies are warranted.

The artificial antibodies produced in this study showed a certain level of neutralizing activity against SARS-CoV-2, and it was more effective when mixed with other antibodies. We selected BCR pairs with high detection frequencies of IgH and IgK chains because BCR analysis from FFPE-derived BCRs could only be performed with bulk-based methods. It is speculated that, not only the BCR pairs we selected and synthesized in this study, but also other combinations of BCRs, may have additional neutralizing activity against SARS-CoV-2. An improved technique is needed to obtain high-quality RNA in adequate quantities from FFPE samples, as not all BCRs can be isolated effectively owing to the limited sample volume and sequencing depth. Although the SARS-CoV-2 neutralizing activity of artificial antibodies synthesized from lung FFPE tissue was superior to vaccine-naïve plasma IgG and similar to 2^nd^ vaccine-treated groups, the SARS-CoV-2 neutralizing effect of the plasma specimens in peripheral blood enhances with the number of mRNA vaccine intakes and the timing of the analysis after vaccination (24). Enriched IgG from peripheral blood may contain a high proportion of antibodies to SARS-CoV-2 due to vaccine injection, but also antibodies to a variety of other antigens. We believe the BCR repertoire analysis in lung tissue from severe cases of COVID-19 may have the highest proportion of antibodies to SARS-CoV-2. Further validation is needed to determine which method-derived antibodies have higher neutralizing effect against SARS-CoV-2.

In conclusion, the BCRs repertoire analysis from the lung tissue where SARS-CoV-2 was present and B-cells were enriched may contribute to produce the effective artificial antibodies or narrow down the candidates of specific BCRs from various BCR repertoires in peripheral blood against various pathogens. Autopsies from raw tissue tend to be avoided in outbreaks of infection where secondary infections is initially suspected, as in the case of SARS-CoV-2. We believe that performing FFPE lung tissue-derived BCR analysis will be an important clue in confronting unknown viral threats in the future.

## Data availability statement

The datasets presented in this study can be found in online repositories. The names of the repositories and accession numbers can be found below: DDBJ DRA - accession number DRA013492; NCBI SRA - accession number PRJDB13054.

## Ethics statement

The studies involving human participants were reviewed and approved by the Research Ethics Committee of Wakayama Medical University (approval no.: 2882, 2961). The patients/participants provided their written informed consent to participate in this study.

## Author contributions

SI, TTs, MKi, TI, KY, MKo, and SH conceived the experiment. KMi, SM, and TK obtained samples and prepared the FFPE block. HM, ST collected whole blood samples from healthy donors with informed consent. YS performed high-throughput sequencing, and SI, TO, MKi, SS, KI, SH developed and performed data analysis. SI, SU, TIt, MKo, KMa, TTo, HY, SH interpreted the data. SI, TTs, MKi and SH wrote the manuscript with contributions from all authors. All authors contributed to the article and approved the submitted version.
