# Supplementary material for: B cell receptor repertoire analysis from autopsy samples of COVID-19 patients
Source: Front Immunol. 2023 Feb 23;14:1034978. doi: 10.3389/fimmu.2023.1034978 (PMC9996338; doi:10.3389/fimmu.2023.1034978)
Supplement: Supplementary file 1 [file DataSheet_1.pdf]

Supplementary Material

1 Supplementary Table 1.

The sequence of primers for single-chain Fc fragment PCR assembly.

Name	Sequence (5’ to 3’)
IGHV1-69-NCoI	ATGGACTGGACCTGGAGGTTCCTCTTTGTGGTGGCAGCAGCTACAGGTGTCCAGT CCCAGGTGCAGCTGGTGCAGTCTGGGGCTGAGGTGAAGAAGCCTGGGTCCTCGGT GAAGGTCTCCTGCAAGGCTTCTGGAGGCACCTTCAGCAGCTATGCTATCAGCTGG GTGCGACAGGCCCCTGGACAAGGGCTTGAGTGGATGGGAGGGATCATCCCTATCT TTGGTACAGCAAACTACGCACAGAAGTTCCAGGGCAGAGTCACGATTACCGCGG ACAAATCCACGAGCACAGCCTACATGGAGCTGAGCAGCCTGAGATCTGAGGACA CGGCCGTGTATTACTGTGCGAGAGACAACGACGATAAGGTGGTAATATATGCACT GGATGTCTGG
IGHV1-69-Vprimer	GGCGGAGGTGGCTCTGGCGGTGGCGGATCGATGGACTGGACCTGGAGGTTC
IGHV1-69-Jprimer	TCAGATCTAACCATGCCAGACATCCAGTGCATATAT
IGKV2-28-EcoRV	ATGAGGCTCCCTGCTCAGCTCCTGGGGCTGCTAATGCTCTGGGTCTCTTCCAGTGG GGATATTGTGATGACTCAGTCTCCACTCTCCCTGCCCGTCACCCCTGGAGAGCCG GCCTCCATCTCCTGCAGGTCTAGTCAGAGCCTCCTGCATAGTAATGGATACAACT ATTTGGATTGGTACCTGCAGAAGCCAGGGCAGTCTCCACAGCTCCTGATCTATTT GGGTTCTAATCGGGCCTCCGGGGTCCCTGACAGGTTCAGTGGCAGTGGATCAGGC ACAGATTTTACACTGAAAATCAGCAGAGTGGAGGCTGAGGATGTTGGGGTTTATT ACTGCATGCAAGCTCTAAAGGCTCCGTGGACGTTC
IGKV2-28-Vprimer	GTCACGAATTCGATTATGAGGCTCCCTGCTCAGCTC
IGKV2-28-Jprimer	AGAGCCACCTCCGCCTGAACCGCCTCCACCGAACGTCCACGGAGCCTTTAGAGCT TGC
IGKV3-20-EcoRV	ATGGAAACCCCAGCGCAGCTTCTCTTCCTCCTGCTACTCTGGCTCCCAGATACCAC CGGAGAAATTGTGTTGACGCAGTCTCCAGGCACCCTGTCTTTGTCTCCAGGGGAA AGAGCCACCCTCTCCTGCAGGGCCAGTCAGAGTGTTAGCAGCAGCTACTTAGCCT GGTACCAGCAGAAACCTGGCCAGGCTCCCAGGCTCCTCATCTATGGTGCATCCAG CAGGGCCACTGGCATCCCAGACAGGTTCAGTGGCAGTGGGTCTGGGACAGACTTC ACTCTCACCATCAGCAGACTGGAGCCTGAAGATTTTGCAGTGTATTACTGTCAGC AGTATGGTAGCTCAATATTCACTTTC
IGKV3-20-Vprimer	ACGAATTCGATATTTATGGAAACCCCAGCGCAGCTTCTC
IGKV3-20-Jprimer	AGAGCCACCTCCGCCTGAACCGCCTCCACCGAAAGTGAATATTGAGCTACCATAC TGC
Linker primer	GGTGGAGGCGGTTCAGGCGGAGGTGGCTCTGGCGGTGGCGGATCG

2 Supplementary Figure.1

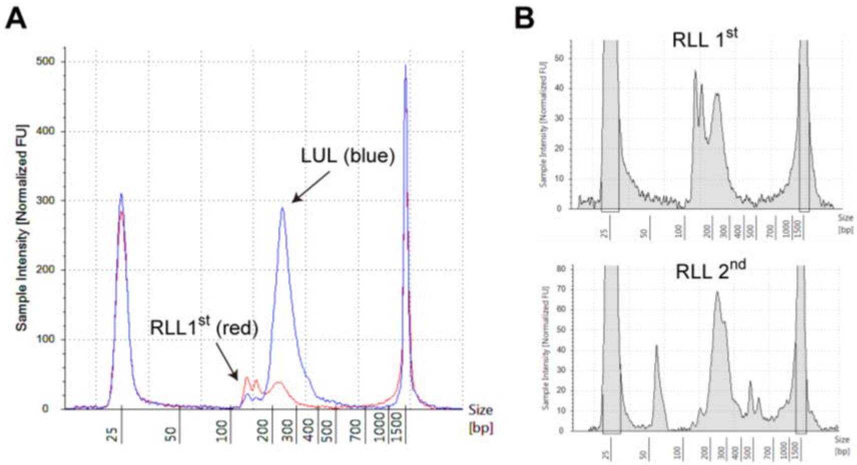

The raw fragmented cDNA data obtained from the Agilent 4200 TapeStation system by using Agilent D1000 Screen Tape Assy. (**A**) The sample intensity in left upper lung lobe (LUL, blue) or right lower lung lobe (RLL, red) is shown. The sequencing libraries was prepared by using bulk BCR analysis, and 1 μl of each sample was measured. (**B**) The graphs indicate the sample intensity in 1st or 2nd trials of RLL. We collected RNA from different slices of RLL in FFPE block, and the sequencing library was prepared twice.

3 **Supplementary Figure 2.**

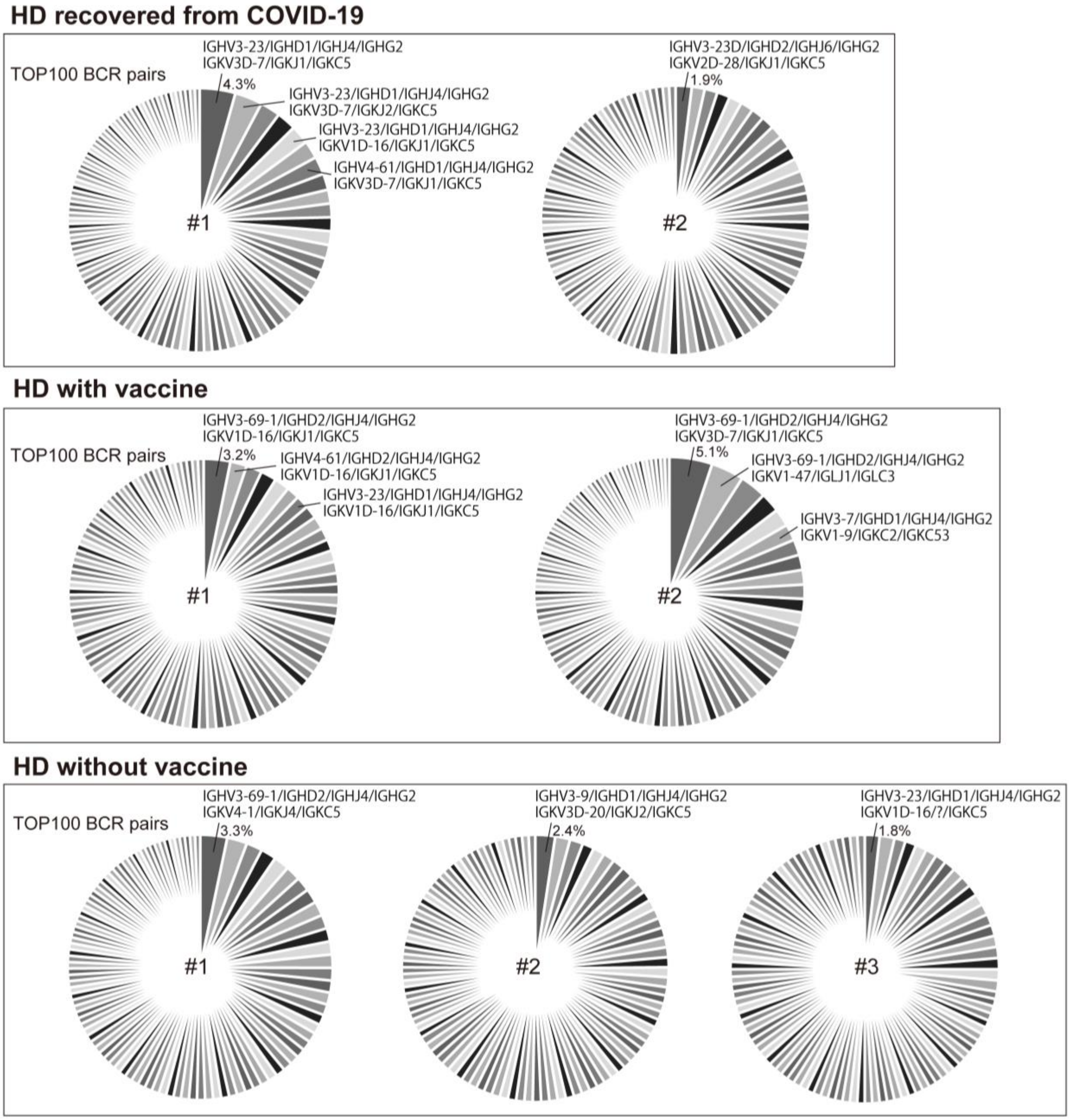

The pie graph of BCR pairs obtained from seven healthy donors. The # in each graph indicates the donor number, and % indicates the ratio of each BCR pair to the TOP100 BCR pairs with high frequently detected. Two HD recovered from COVID-19, two HD with vaccine, and three HD without vaccine treatment group is shown. The raw fastq data is deposited in DRA013491.

4 Supplementary Table 2.

Confirmation of SARS-CoV-2 IgG by iFlash3000

Samples	N(+S) IgG (AU/mL)	S1 IgG (AU/mL)
HD-COVID-19#1	55.78	34.85
HD-COVID-19#1	10.58	22.47
HD without vaccine#1	1.29	0.37
HD without vaccine#2	0.7	0.43
HD without vaccine#3	2.97	0.46
HD with vaccine#1	0.34	1136.31
HD with vaccine#2	0.23	1353.32

Supplementary Figure 3.

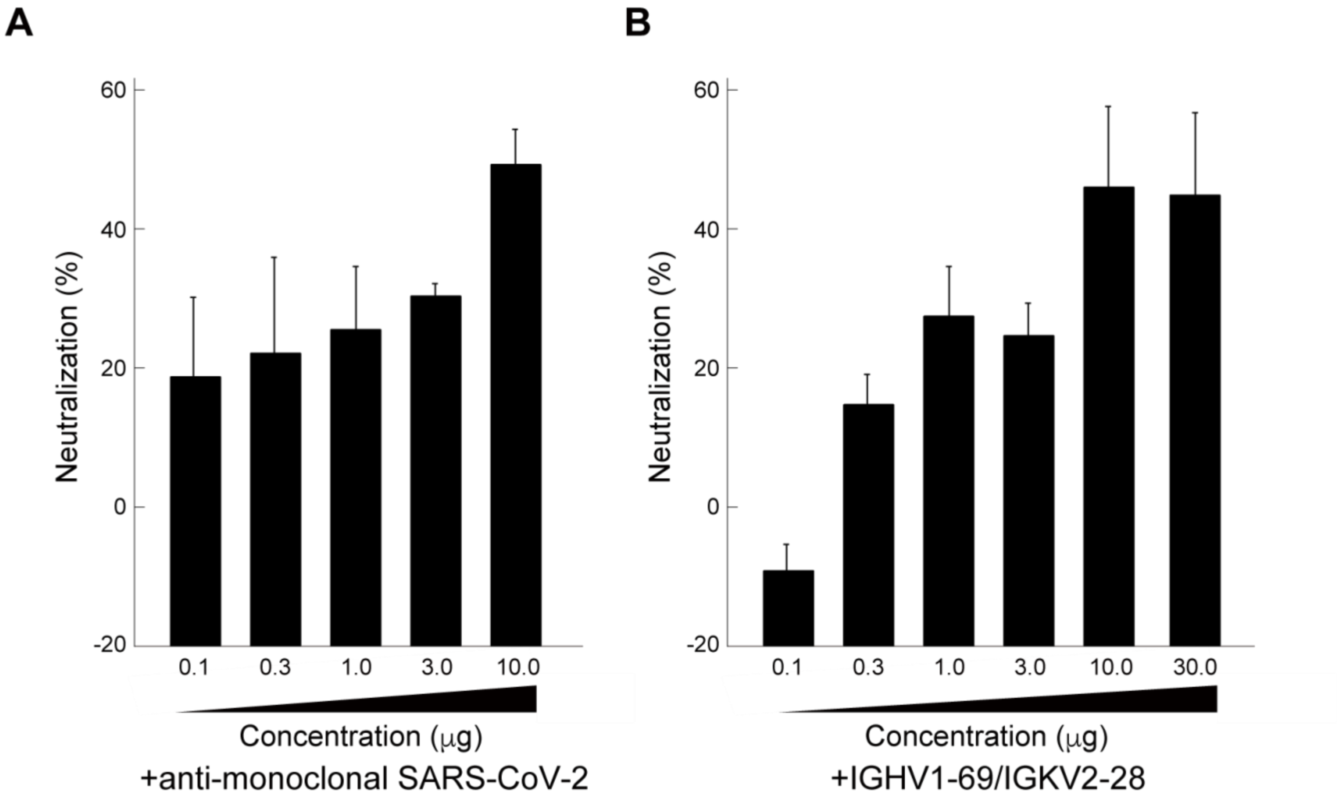

(**A**) The graph indicates the dose-dependent effects for number of plaques in positive control group; the % of neutralization in 100 ng, 300 ng, 1 mg, 3 μg and 10 μg of SARS-CoV-2 S protein monoclonal antibody are shown. The bars indicate mean ± standard error (S.E.), and number of samples is two in each condition. (B) The dose-dependent neutralization effects (%) in *IGHV1-69/IGHV2-28* treatment groups. Live SARS-CoV-2 (TY/WK521) was reacted with 100 ng, 300 ng, 1 μg, 3 μg, 10 μg and 30 μg of artificial antibody was applied to the VeroE6/TMPRSS2 cells. The bars indicate mean ± S.E., and number of samples is two in each condition.

6 Supplementary Figure 4.

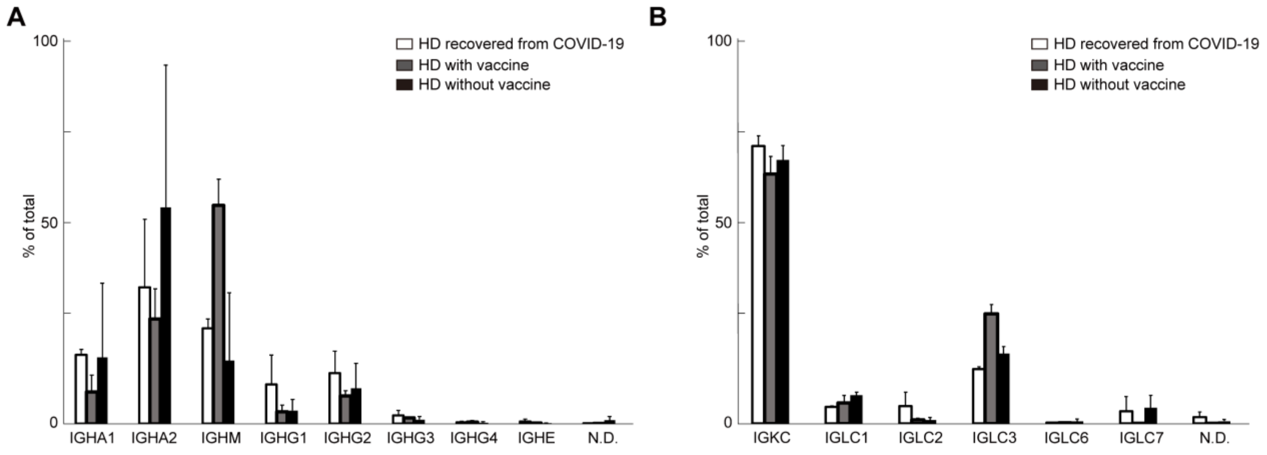

The distribution of the type of BCR heavy or light chains obtained from scBCR-seq analysis in each group. (**A**) The graph indicates the % of IGHA1-2, IGHM, IGHG1-4, IGHE, not detected (N.D.) for total IgH chains. We could not identify the type of IgG (N.D.) in some BCR repertoire. HD recovered from COVID-19 (white bar), HD with vaccine (grey), and HD without vaccine treatment (black) groups. The bars indicate mean ± standard deviation (S.D.). (**B**) The graph shows the % of IGKC, IGLC1-3, 6, 7, and N.D. for total BCR light chains. The bars indicate mean ± standard deviation (S.D.).
